# 4371 The Role of B Cells in Keloid Formation

**DOI:** 10.1017/cts.2020.97

**Published:** 2020-07-29

**Authors:** Jaclyn B Anderson, Alexander B Harrant, Nalu Navarro-Alvarez, Zhaohui Wang, Adrie van Bokhoven, Whitney High, Tae W Chong, Christene A. Huang

**Affiliations:** 1 University of Colorado at Denver

## Abstract

OBJECTIVES/GOALS: Recent studies indicate B cells are involved in dermal fibroblast activation and collagen deposition in the skin. However, B cell distribution in epidermal and dermal layers is unknown. Here, We aim to characterize the distribution of B cells residing in normal skin and keloidal scars. METHODS/STUDY POPULATION: One abdominal normal skin sample and two keloid samples (ear and shoulder) were obtained from the University of Colorado Biorepository Core Facility and from the Plastic Surgery Clinics. Five micron sections from formalin-fixed paraffin-embedded samples were prepared for multiplex fluorescence immunohistochemistry by the Human Immunology & Immunotherapy Initiative. We stained for CD20+, CD19+, and DAPI. Slides were imaged using Vectra®3 scanning system from PerkinElmer. Images were analyzed in InForm®Tissue Finder, phenotpr, phenoptrReports by Akoya biosciences. RESULTS/ANTICIPATED RESULTS: We found a significant increase in the percentage of CD20+ and CD19+ B cells in keloid skin compared to normal skin tissue (14.50% and 14.20% vs 6.47% and 7.56% of the total cells), respectively. Interestingly, we found that in the epidermis of keloid skin CD20+ cell were more abundant (14.46%) whereas in the epidermis normal skin CD20+ cells were less predominant (5.14%). In the dermis of keloid skin, CD20+ and CD19+ were in equal proportions (13%) whereas in normal skin CD19+ cells were more predominant (10.44%) compared to CD20+ cells (7.04%). Dual positive B cells, CD19+/CD20+ cells, were more abundant in keloid dermis (11.06%) compared to normal skin dermis (1.24%). DISCUSSION/SIGNIFICANCE OF IMPACT: B cells are involved in fibroblast activation in diseases such as scleroderma and rheumatoid arthritis. With the increase of CD19+/CD20+ B cells in keloids, the role of B cells in keloid pathogenesis warrants further study. CD27 staining may determine if these are activated or follicular B cells.

